# Incidence and predictors of mortality among children co-infected with tuberculosis and human immunodeficiency virus at public hospitals in Southern Ethiopia

**DOI:** 10.1371/journal.pone.0253449

**Published:** 2021-06-30

**Authors:** Zinabu Dawit, Sintayehu Abebe, Samuel Dessu, Molalegn Mesele, Serekebirhan Sahile, Desalegn Ajema

**Affiliations:** 1 Department of Nursing, Arba Minch College of Health Sciences, Arba Minch, Southern Ethiopia; 2 School of Public Health, College of Medicine and Health Sciences, Arba Minch University, Arba Minch, Southern Ethiopia; 3 Department of Public Health, College of Medicine and Health Sciences, Wolkite University, Wolkite, Southern Ethiopia; 4 Department of Midwifery, College of Health Sciences and Medicine, Wolaita Sodo University, Wolaita, Southern Ethiopia; The University of Georgia, UNITED STATES

## Abstract

**Background:**

Tuberculosis and human immune deficiency virus co-infections remained the most common cause of child mortality for the last ten years. Globally, 1.2 million cases of tuberculosis occurred in patients living with HIV/AIDS, of which 1.0 million cases occurred in children. The public health impact of tuberculosis and human immune deficiency virus co-infection among children is high in developing countries and Sub-Saharan Africa accompanied three fourth of the global burden. However, there are limited studies that assess the incidence and predictors of mortality among tuberculosis and human immune deficiency virus co-infected children in Ethiopia.

**Methods:**

A facility-based retrospective cohort study was conducted at Public hospitals in Southern Ethiopia with a total of 286 randomly selected records of ART enrolled children from 1st January 2009 to 31^st^December 2018. Data were entered into Epi Data version 3.1 and exported to STATA version 14 for analysis. Bivariate and multivariable Cox proportional hazards model was fitted to identify the predictors of mortality. Variables that had a p-value<0.05 at 95%CI in the multivariable cox proportional hazard model were considered as statistically significant.

**Results:**

A total of 274 tuberculosis and human immunodeficiency virus co-infected children’s records were reviewed. The incidence of mortality among tuberculosis and human immunodeficiency virus co-infected children was 17.15 per 100 children. The overall incidence density rate of mortality was 2.97(95%CI: 2.2, 3.9) per 100 child year of observation and being anemic (AHR: 2.6; 95%CI: 1.28, 5.21), not initiating isoniazid prophylaxis therapy (AHR: 2.8; 95%CI: 1.44, 5.48), developing extrapulmonary tuberculosis (AHR: 5.7; 95%CI: 2.67, 12.56) and non-adherence (AHR: 5.2; 95%CI: 2.19, 12.39) were independent predictors of mortality.

**Conclusion:**

Mortality rate was high among TB/HIV co-infected children at the public hospitals in Southern Ethiopia. Extra-pulmonary tuberculosis, anemia, non-adherence, and isoniazid preventive therapy use were statistically significant predictors of mortality among TB/HIV co-infected children. Therefore, extra pulmonary tuberculosis, and anemia should be closely monitored to increase their adherence as well as they should be provided with isoniazid preventive therapy.

## Background

Human immunodeficiency virus (HIV) infection is the greatest risk factor for acquiring Tuberculosis (TB) infection and developing the disease [1, 2]. Tuberculosis (TB) enhances human immunodeficiency virus (HIV) replication by accelerating the natural evolution of HIV infection; it is the leading cause of sickness and death of people living with HIV [2]. Human immunodeficiency virus and Tuberculosis (TB/HIV) co-infection is “bidirectional and synergistic in which HIV promotes the progression of latent tuberculosis infection to disease, and tuberculosis accelerates the progression of HIV disease to its advanced stage [3–5].

Tuberculosis and human immune deficiency virus co-infections remained the most common cause of child mortality for the last ten years. Globally, 1.2 million cases of tuberculosis occurred in patients living with HIV/AIDS, of which 1.0 million cases occurred in children [6]. It mostly affects the lungs (pulmonary TB) but can affect other sites as well [7]. Relatively, a small proportion of people infected with Mycobacterium tuberculosis will develop TB disease but the probability of developing TB is much higher among people infected with HIV [8].

The double burden of tuberculosis (TB) and human immunodeficiency virus (HIV) is one of the major global health challenges of the 21^st^ century [9]. TB is the leading immune-suppressing infection and the commonest cause of death among HIV-infected children [10]. The burden of tuberculosis and human immunodeficiency virus co-infection is particularly high in developing countries and approximately three-fourths of the global TB/HIV co-infection observed in Sub-Saharan Africa including Ethiopia [6]. TB and HIV work together to suppress the immunity of the patients and thereby, shorten the lifespan if early treatment is not initiated [11, 12].

The Ethiopian Federal HIV and AIDS Prevention and Control Office estimated that the single National HIV/AIDS is among the top ten high burden counties with an incidence rate of 341/100,000 of which 31% of TB patients are living with HIV [13]. Still now TB/HIV co-infection is the leading cause of death in people living with HIV/AIDS [14].

Ethiopia is one of the 30 high burden countries and has been classified as having high burdens of TB/HIV co-infection. The country is striving to reduce the magnitude of TB and HIV disease in line with the strategies to achieve the Sustainable Development Goals (SDG) [2]. However, the problem remains significant, particularly in HIV-positive children. The cause of mortality among TB/HIV co-infected children is multi-factorial which includes diagnosis age, nutritional status, immunity status, and hemoglobin levels at ART initiation [15]. Therefore; this study was aimed to determine the incidence and predictor’s of mortality among children co-infected with TB/HIV at public hospitals in Southern Ethiopia.

## Methods and materials

### Study design and setting

A facility-based retrospective cohort study was conducted in seven selected public hospitals in Southern Ethiopia from the records enrolled from1st January 2009 to 31st December 2018. Those hospitals are located in the southern part of Ethiopia. All TB/HIV co-infected children under 15 years of age who ever enrolled in pediatrics ART clinics were the source populations. The starting point is from entry to children who were TB/HIV co-infected service from 1st January 2009 to 31st December 2018 and the endpoint was death or loss to follow up or transferred to another.

### Study population and sampling technique

The source population was all TB/HIV co-infected children under 15 years of age who enrolled in a treatment program in public hospitals in Southern Ethiopia. The sample size was calculated based on estimation for the assessment of survival time using Epi info version 7 in considering the following assumptions: 95% CI, power of 80%, the ratio of unexposed to exposed 1:1 and Parameters: P_1_: is the proportion of exposed with the outcome 11.81, P_2_: is the proportion of non-exposed with the outcome 2.21 and 5% marginal error. Finally, by using 10% for incompleteness the sample size was 286. The Sample was allocated proportionally for the seven selected facility and records were selected randomly.

### Data collection procedure and data quality control

The sources of data for this study were the Pre-ART register, the ART register, and the patients’ ART follow-up and medical charts. In those registers and follow-up charts, clients’ socio-demographic, clinical, and laboratory information, treatments being provided, the follow-up status of each client were recorded. Data was collected from client charts using a structured checklist for records review developed from the registers and follow-up charts. Twenty-one data collectors who are health professionals and working in pediatric ward were recruited for data collection after getting training on the tool.

### Study variables and data analysis

The outcome variable is time to death from enrolment to the CART program. The survival time is measured as the time period between the date of enrolment and date of death and it is dichotomized as death and censored. The censored cases include the alive patients, defaulters, and transferred-outs. Data were cleaned, coded, and entered into Epi Data version 3.1 and exported to STATA version 14 for analysis. Bivariate analysis was carried out to determine the association between the dependent variable and the explanatory variables. Both Crude hazard ratio (CHR) and adjusted hazard ratio(AHR) together with the corresponding 95% confidence interval and P-value were used to assess the strength of association and statistical significance. The Kaplan Meier survival curve together with the log-rank test was fitted to determine the survival time. Variables which had p-value <0.25 in bivariate analysis were considered as a candidate for multivariable analysis and variables which had p-value <0.05 in multivariable cox regression analysis were considered as statistically significant. The backward stepwise regression method was applied.

### Ethical consideration

Ethical clearance was obtained from the institutional review board (IRB) of the Arba Minch University College of medicine and health sciences. In addition; a permission letter was obtained from the Arba Minch University and public hospital administrations. HIV care clinics’ focal persons were informed about the objective and significance of the study prior to the data collection. Appropriate measures were applied to ensure the confidentiality of the data. All data were fully anonymized before we accessed them and the ethical review board waived the requirement for informed consent.

## Results

### Socio-demographic characteristics

A total of 286 TB/HIV co-infected children’s charts were reviewed. Of these, 12(4.19%) were excluded from the analysis due to incomplete data. Therefore, 274 TB/HIV co-infected children were included in the analysis. The mean age of the study participants was 8.9(±3.5 SD) years. Eight (2.9%) of the children were under one year of age and more than half (66.8%) of children’s caregivers were between the age group of (25–34) years with a median age of 30 (IQR (27.72–34) years. Regarding the sex of the children, nearly half (50.7%) were males. More than three fourths (77.4%) of the respondent were urban residents and 254 (92.4%) children were lives together with their parents. Approximately, two-thirds (70.1%) of the children’s caregivers were HIV positive in their HIV status. Regarding the family size of the children, 39(14.8%), 196(74.5%) and 28(10.6%) of the children had less than two, 3–4 and more than four family size respectively (**Table 1**).

**Table 1:** Socio-demographic characteristics of TB/HIV co-infected children at public hospitals in Southern Ethiopia, 2020.

Characteristics	Categories	Survival Status				
		Total N (%) N = 274	N PY	Death N (%) N = 47	Censored N (%) N = 227	IDR
Age (year)	<1	11(4.0)	58.8	2(4.3)	9(4.0)	3.40
	1–5	18(6.6)	109	4(8.5)	14(6.2)	3.66
	6–10	113(41.2)	739	18(38.3)	95(41.9)	2.43
	11–14	132(48.2)	674.5	23(48.9)	109(48.0)	3.40
Sex	Male	139(50.7)	747.7	22(46.8)	117(51.5)	2.97
	Female	135(49.3)	833.6	25(53.2)	110(48.5)	2.99
Age of caregiver	15–24	35(12.8)	220	6(12.8)	29(12.8)	2.27
	25–34	183(66.8)	1050	32(68.1)	151(66.5)	3.06
	35–44	40(14.6)	216.3	4(8.5)	36(15.9)	1.86
	>44	16(5.8)	95	5(10.6)	11(4.8)	5.26
Residence	Urban	212(77.4)	1232.5	32(68.1)	180(79.3)	2.61
	Rural	62(22.6)	348.8	15(31.9)	47(20.7)	4.30
Caregiver of the child	Mother	217(79.2)	1291.5	37(78.7)	180(79.3)	2.87
	Father	28(10.2)	159.3	2(4.3)	26(11.5)	1.25
	Stepparents	11(4.0)	28.5	2(4.3)	9(40)	7.01
	Sibling	18(6.6)	102	6(12.8)	12(5.3)	5.94
Caregiver HIV status	Positive	192(70.1)	1156.8	33(70.2)	159(70)	2.86
	Negative	39(14.2)	180.5	6(12.8)	33(14.5)	3.34
	Unknown	43(15.7)	244	8(17.0)	35(15.4)	3.27

*PY*: *Person years of observation*, *IDR*: *Incidence density rate*

### Clinical characteristics

Among the total 274 children, 165(60.2%) of children had baseline HIV WHO clinical stage (III and IV). The eligibility criteria for initiation of HAART were both CD4+ cell count or percent and WHO clinical stage. More than one fifths (21.2%) of the children were initiated HAART based on both WHO clinical staging and CD4+. More than one fourths (28.1%) of children had experienced initial regiment change during their follow-up time.

More than one tenths (19.5%) of the children face treatment failure, among them 6.4% were dead. The median hemoglobin level of the respondents was 11 (IQR: 10, 12). Hence; 16.1% of the children were Anemic at the baseline. Regarding prophylaxis, more than three fourths (79.9) of the children were initiated cotrimoxazole and nearly three fourths (74.8%) of the children were initiated isoniazid preventive therapy. Among the nutritional problems, 2.6% and 2.2% of the children develop underweight and stunting respectively. More than half (52.2%) of the children develop TB at the pre-ART period (**Table 2**).

**Table 2:** Clinical characteristics of TB/HIV co-infected children at public hospitals Southern Ethiopia, 2020.

Characteristics		Total N (%) N = 274	N PY	Death N% N = 47	Censored N (%) N = 227	IDR
Baseline WHO stage	Mild (I & II)	109(39.8)	710.5	18(38.3)	91(40.1)	2.55
	Advanced (III&IV)	165(60.2)	870.8	29(61.7)	136(59.9)	3.33
ART Eligibility criteria	CD4+ cell	43(15.7)	233.5	12(25.5)	31(13.7)	5.13
	WHO stage	169(61.7)	1038	26(55.3)	143(63.0)	2.52
	Both	58(21.2)	303.8	8(17.0)	50(22.0)	2.60
	Test and treat	4(1.5)	6	1(2.1)	3(1.3)	16.6
Initial ART regimen	4a = D4T-3TC-NVP	143(52.2)	964.5	20(42.6)	123(54.2)	2.08
	4b = d4T-3TC-EFV	6(2.2)	34.8	1(2.1)	5(2.2)	2.87
	4c = AZT-3TC-NVP	26(9.5)	157.6	7(14.9)	19(8.4)	4.44
	4e = TDF-3TC-EFV	75(27.4)	301.4	16(34.0)	59(26.0)	5.34
	4f = AZT+3TC+LPV	18(6.6)	104	2(4.3)	16(7.0)	1.92
	4g = ABC+3TC+LPV	6(2.2)	19	1(2.1)	5(2.2)	5.26
Initial regimen change	Yes	77(28.1)	520.2	14(29.8)	63(27.8)	2.70
	No	197(71.9	1056.1	33(70.2)	164(72.2)	3.12
Reason for regimen change	Side effect	38(49.4)	221.9	5(10.6)	33(14.5)	2.27
	Treatment failure	15(19.5)	115.5	3(6.4)	12(5.3)	2.59
	TB	17(22.1)	77	4(8.5)	13(5.7)	5.19
	Stock out	7(9.1)	30	2(4.3)	5(2.2)	6.66
	Not change regimen	197(71.9)	1136.9	33(70.2)	164(72.2)	2.91
Treatment failure	Yes	15(5.5)	92	3(6.4)	12(5.3)	3.26
	No	259(94.5)	1489.3	44(93.6)	215(94.7)	2.96

Among the records of children, 3(1.1%) of them developed an immunological failure and among them, 1(2.1%) was dead. Among the records reviewed, 269(97.4%) did not face virologic failure. Among the records that face virologic failure, one (2.1%) was the record of a dead child. Nearly three-fourths (74.8%) of the records of children, were records of children who use Isoniazid preventive therapy and 219 (79.9%) CPT (Table 3).

**Table 3:** Medication and disease related characteristics of TB/HIV co-infected children at public hospitals Southern Ethiopia, 2020.

Variables	Category	Total N (%) N = 274	N PY	Death N% N = 47	Censored N (%) N = 227	IDR
Immunologic failure	Yes	3(1.1)	18	1(2.1)	2(0.9)	5.55
	No	271(98.9)	1563.3	46(97.9)	225(99.1)	2.95
Virologic failure	Yes	5(1.5)	30	1(2.1)	4(1.8)	3.33
	No	269(97.4)	1551.3	46(97.9)	223(98.2)	2.97
Clinical failure	Yes	7(2.6)	41	3(6.4)	4(1.8)	7.31
	No	267(97.4)	1540.3	44(93.6)	223(98.2)	2.86
Isoniazid	Yes	205(74.8)	1243	16(34.0)	189(83.3)	1.28
	No	69(25.2)	338.3	31(66.0)	38(16.7)	9.35
CPT	Yes	219(79.9)	1302.3	20(42.6)	199(87.7)	1.54
	No	55(20.1)	279	27(57.4)	28(12.3)	9.71
Weight for age	Normal	267(97.4)	1541.3	46(97.9)	221(97.4)	2.97
	Underweight	7(2.6)	30	1(2.1)	6(2.6)	3.33
Height for age	Normal	268(97.8)	1548.3	46(97.9)	222(97.8)	2.98
	Stunting	6(2.2)	33	1(2.1)	5(2.2)	3.03
Functional status	Working	208(75.9)	1271.5	34(72.3)	174(76.7)	2.68
	Ambulatory	36(13.1)	167.2	7(14.9)	29(12.8)	4.18
	Bedridden	30(10.9)	142.6	6(12.8)	24(10.6)	4.23
Adherence	Adherence	193(70.4)	1186.5	9(19.1)	184(81.1)	0.75
	Non-adherence	81(29.6)	394.8	38(80.9)	43(18.9)	9.77
Type of TB	PTB	187(68.2)	1153.3	9(19.1)	178(78.4)	0.78
	EPTB	87(31.8)	428	38(80.9)	49(21.6)	8.92
Period of TB diagnoses	PRE ART	143(52.2)	793	27(57.4)	116(51.1)	3.43
	ART	131(47.8)	788.3	20(42.6)	111(48.9)	2.53
Hgb level at TB diagnose	<10 mg/dl	44(16.1)	217	27(57.4)	17(7.5)	12.5
	> = 10 mg/dl	230(83.9)	1364.3	20(42.6)	210(92.5)	1.47

*PY*: *Person years of observation*, *IDR*: *Incidence density rate*

### Incidence of mortality

In this study, 274 children were followed for a total of 1581.3 child years of observation. The minimum and maximum follow-up periods were one and ten years respectively with the median follow-up period of six(IQR: 3–8) years. Therefore; the incidence of mortality among TB/HIV co-infected children was 17.15% and the incidence density rate was 2.97 per 100 child year observation (95%CI: 2.2, 3.9).

### The survival status of TB/HIV co-infected children

In this study, 3.28% of the children were dead at the end of the first year. In addition, 5.10% and 9.12% of the children were died at the end of the first two years and first five years respectively. The cumulative proportion of survival at the end of the first year, third years, fifth years, and the tenth year was 96 (95% CI: 93, 98), 92 (95% CI; 87, 94), 89 (95% CI; 84, 92) and 56 (95% CI; 39,70) respectively **(Table 4)**.

**Table 4:** Life table showing the cumulative survival probability among TB/HIV co-infected children at public hospitals in Southern Ethiopia, 2020.

Year	No of children at start	Withdrawn during years	At risk	Deaths	Prob. of death	Prob. of surviving year	Cumulative probability of surviving	95% CI
**1**	274	36	256	9	0.04	0.96	0.96	0.93,0.98
**2**	229	12	223	5	0.02	0.98	0.94	0.90,0.96
**3**	212	11	206.5	5	0.02	0.98	0.92	0.87,0.94
**4**	196	11	190.5	3	0.02	0.98	0.91	0.86,0.93
**5**	182	14	175	3	0.02	0.98	0.89	0.84,0.92
**6**	165	29	150.5	7	0.05	0.95	0.85	0.79,0.89
**7**	129	25	116.5	4	0.03	0.97	0.82	0.75,0.86
**8**	100	36	82	4	0.05	0.95	0.78	0.70,0.83
**9**	60	26	47	3	0.06	0.94	0.73	0.63,0.80
**10**	31	27	17	4	0.23	0.77	0.56	0.39,0.70

**Fig 1:**
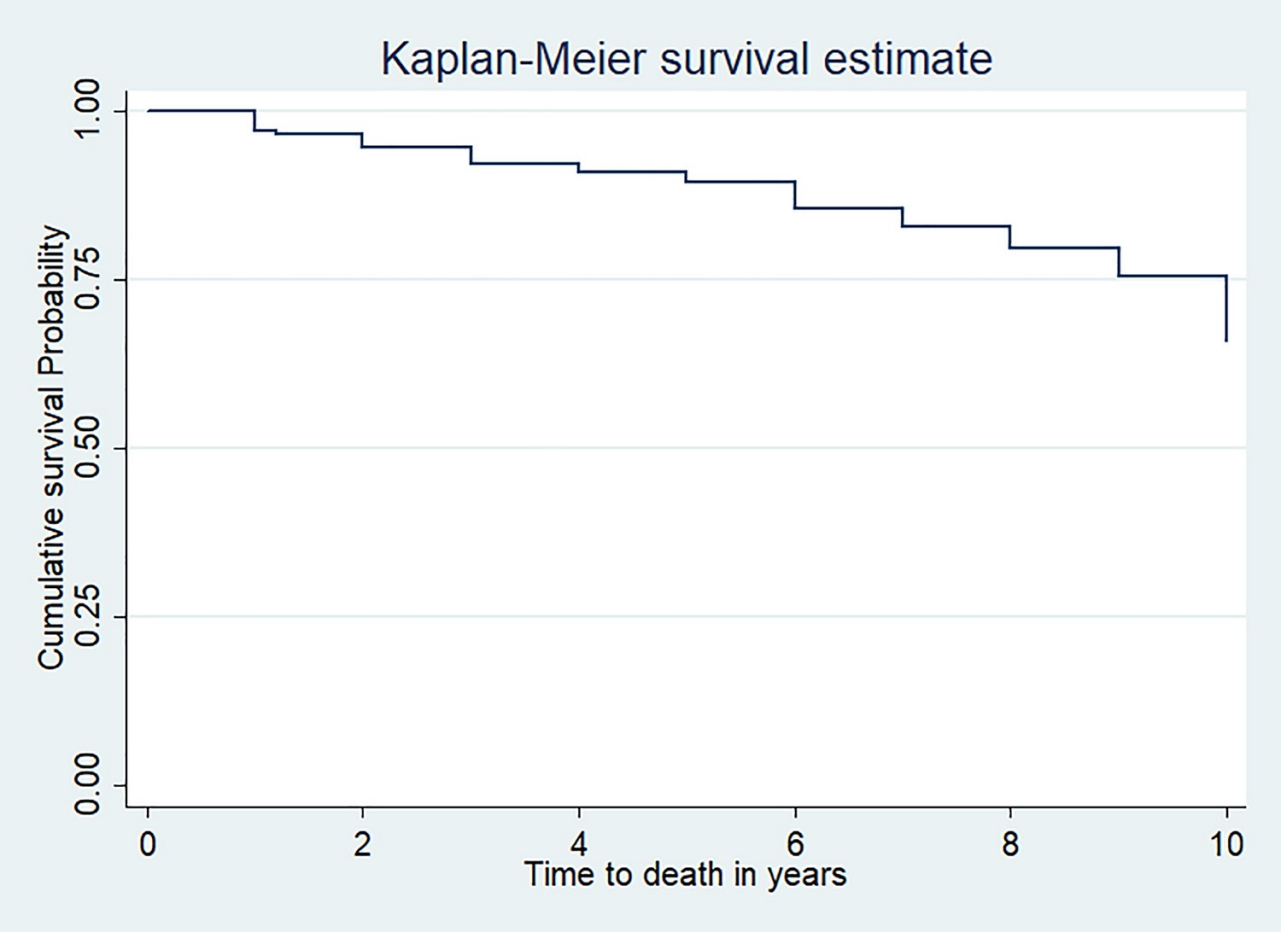
Kaplan Meier curve of survival proportion for TB/HIV co-infected children at public hospitals in Southern Ethiopia, 2020.

In this study, 197(71.9%) children were alive at the end of the follow-up period. In addition; 47(17.1%), 17(6.2%) and 13(4.7%) children were dead, lost to follow up, and transferred out respectively. The overall mean survival of TB/HIV co-infected children was 8.8 year (95%CI: 8.5, 9.19) (**Fig 1**).

### The log-rank estimate of mortality among TB/HIV Co-infected children

The log-rank test estimate revealed that the survival pattern of mortality among TB/HIV Co-infected children was significantly varied among the covariates. The Kaplan Meier survival curve together with the log-rank test shows the effect of each variable on mortality of TB/HIV Co-infected children. Type of TB, Cotrimixazole initiation, Isoniazid preventive therapy initiation, and Hgb level at TB diagnosis were the variables that had a significant effect on mortality among TB/HIV Co-infected children (**Table 5**).

**Table 5:** Log rank estimate of variables among TB/HIV co-infected children at public hospitals in Southern Ethiopia, 2020.

Variables	Log-rank test estimate
Age(year)	X^2^_=_ 1.13, p-value = 0.77
Sex of respondents	X^2^ = 0.001 p-value = 0.995
Mother HIV status	X^2^ = 2.30,p-value = 0.317
Relationship to the child	X^2^ = 6.93, p-value = 0.74
Residence	X^2^ = 3.54,p-vaue = 0.60
Place of health service utilization	X^2^ = 1.17,p-value = 0.27
Family size	X^2^ = 0.28,p-value = 0.86
Age of caregiver	X^2^ = 2.44,p-value = 0.48
WHO clinical stage	X^2^ = 1.03,p-value = 0.30
HIV status of the caregiver	X^2^ = 0.61,p-value = 0.97
Initial regimen	X^2^ = 10.42,p-value = 0.06
Treatment failure	X^2^ = 0.037,p-value = 0.84
Immunologic failure	X^2^ = 0.49,p-value = 0.48
Virologic failure	X^2^ = 0.003,p-value = 0.95
Clinical failure	X^2^ = 2.52,p-value = 0.112
Weight for age	X^2^ = 0.086,p-value = 0.77
Height for age	X^2^ = 0.015,p-value = 0.090
Adherence to ART	X^2^ = 84.80,p-value = 0.0001
Type of TB	X^2^ = 73.53, p-value = 0.0001
Cotrimoxazole	X^2^ = 52.28, p-value = 0.0001
Isoniazid preventive therapy	X^2^ = 56.32, p-value = 0.0001
Functional status	X^2^ = 2.17,p-value = 0.337
Hgb level at TB diagnose	X^2^ = 86.23, p-value = 0.0001

### Comparison of survival probability among categories of variables

**Fig 2:**
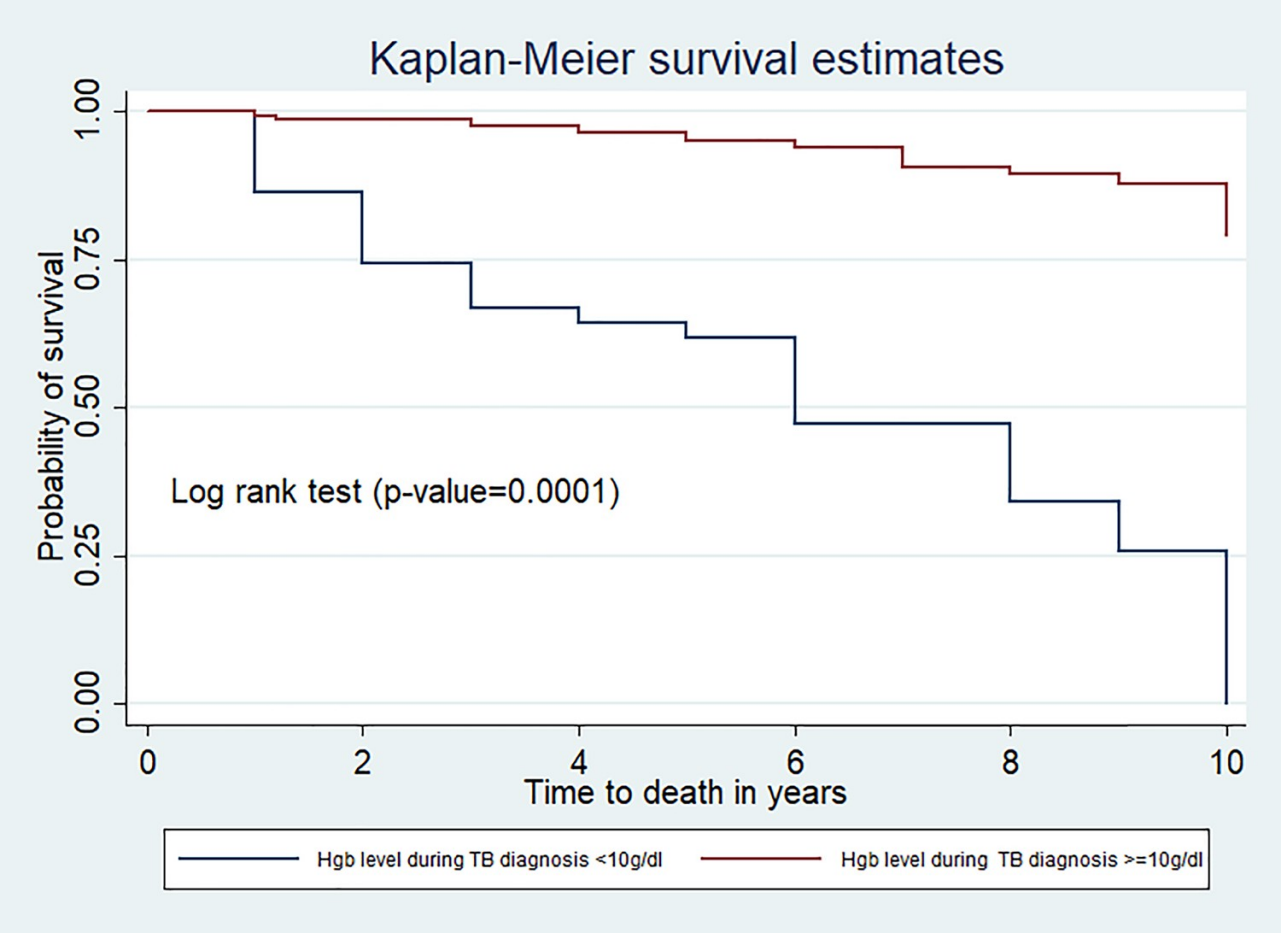
Kaplan Meier survival estimates of hemoglobin level at TB diagnosis among TB/HIV co-infected children at public hospitals, Southern Ethiopia.

The mean survival time for those who had hemoglobin level less than 10 g/dl at the TB diagnosis period was 6.0(95%CI; 5.0,7.15) years and it was 9.48(95%CI: 9.28, 9.73) years for the children who had hemoglobin level greater than 10g/dl at the diagnosis of TB (p-value,<0.0001) (**Fig 2**).

**Fig 3:**
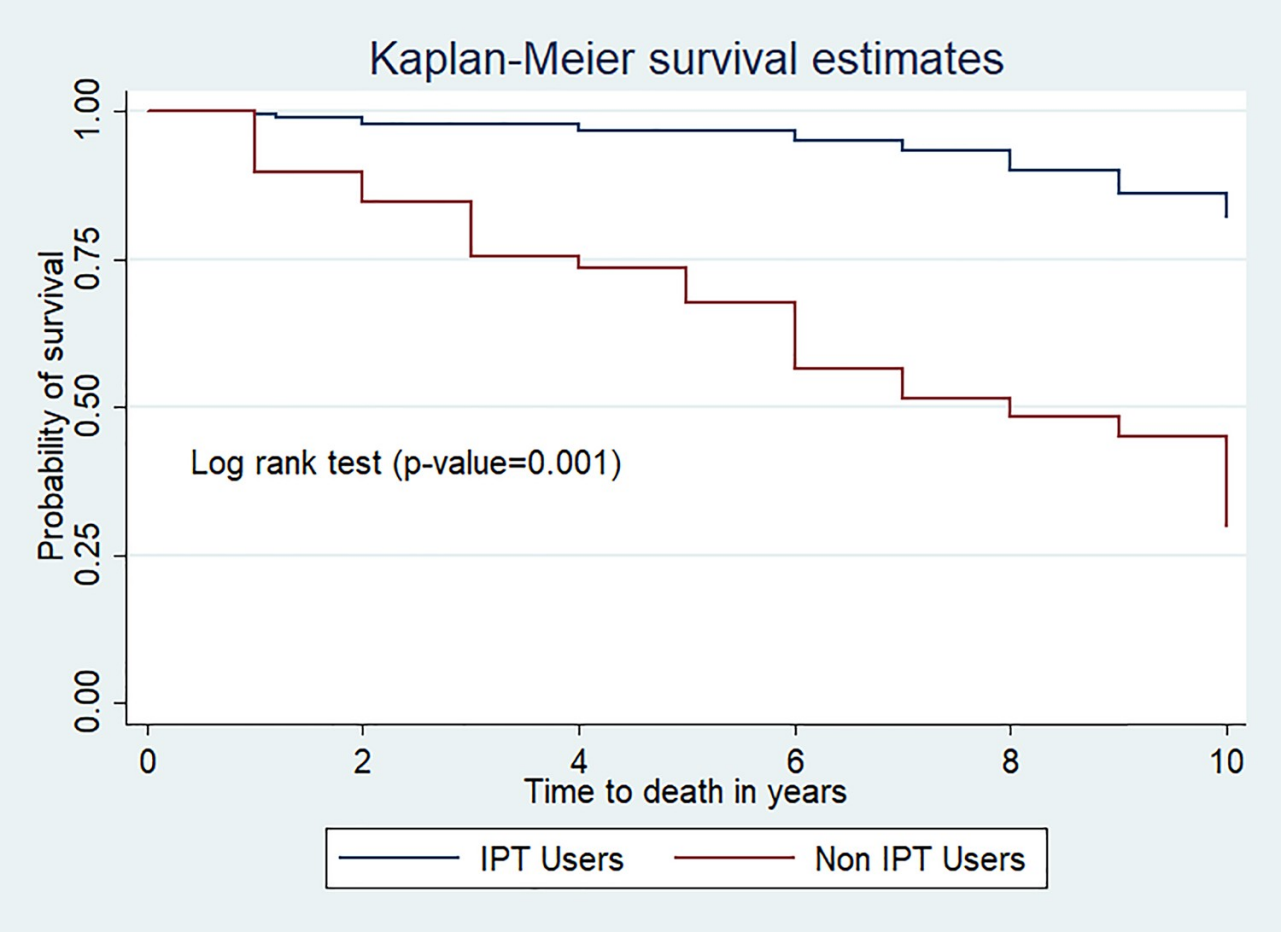
Kaplan Meier survival curve of IPT use among TB/HIV co-infected children at public hospitals, Southern Ethiopia, 2020.

The mean survival time of those who use CPT was 9.52 (95%CI; 9.27, 9.77) year while it was 6.92(95%CI; 6.0, 7.81) year for those who did not use CPT (p-value<0.001) (**Fig 3**).

**Fig 4:**
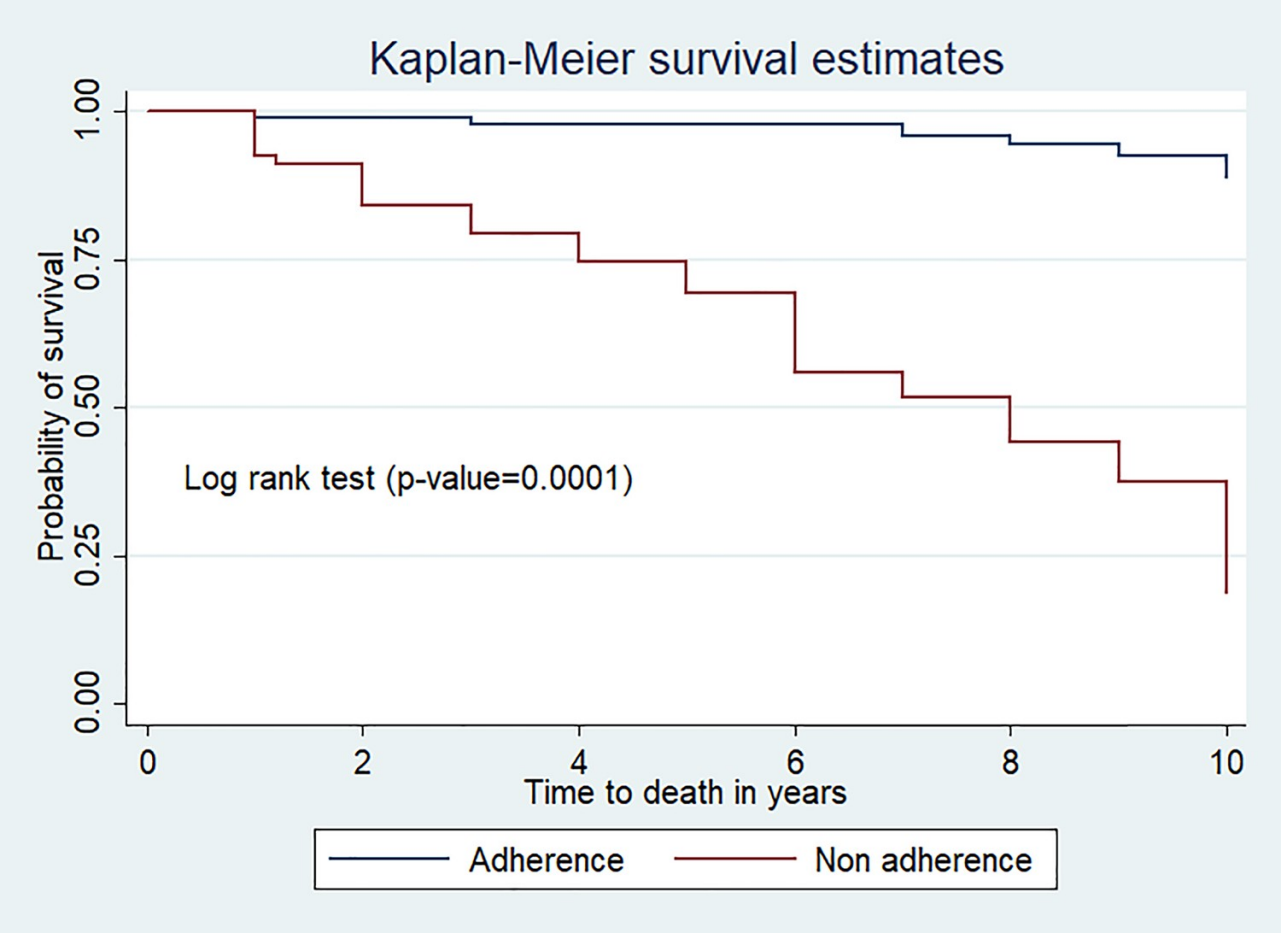
Kaplan Meier survival curve of ART adherence level among TB/HIV co-infected children at seven public hospitals, Southern Ethiopia.

The mean survival time of children co-infected with TB/HIV who had adherence to ART drugs was high 9.71(95%CI; 9.50, 9.92) year as compared to non-adherence 6.8(95%CI; 6.09, 7.6) year (p-value<0.0001) (**Fig 4**).

**Fig 5:**
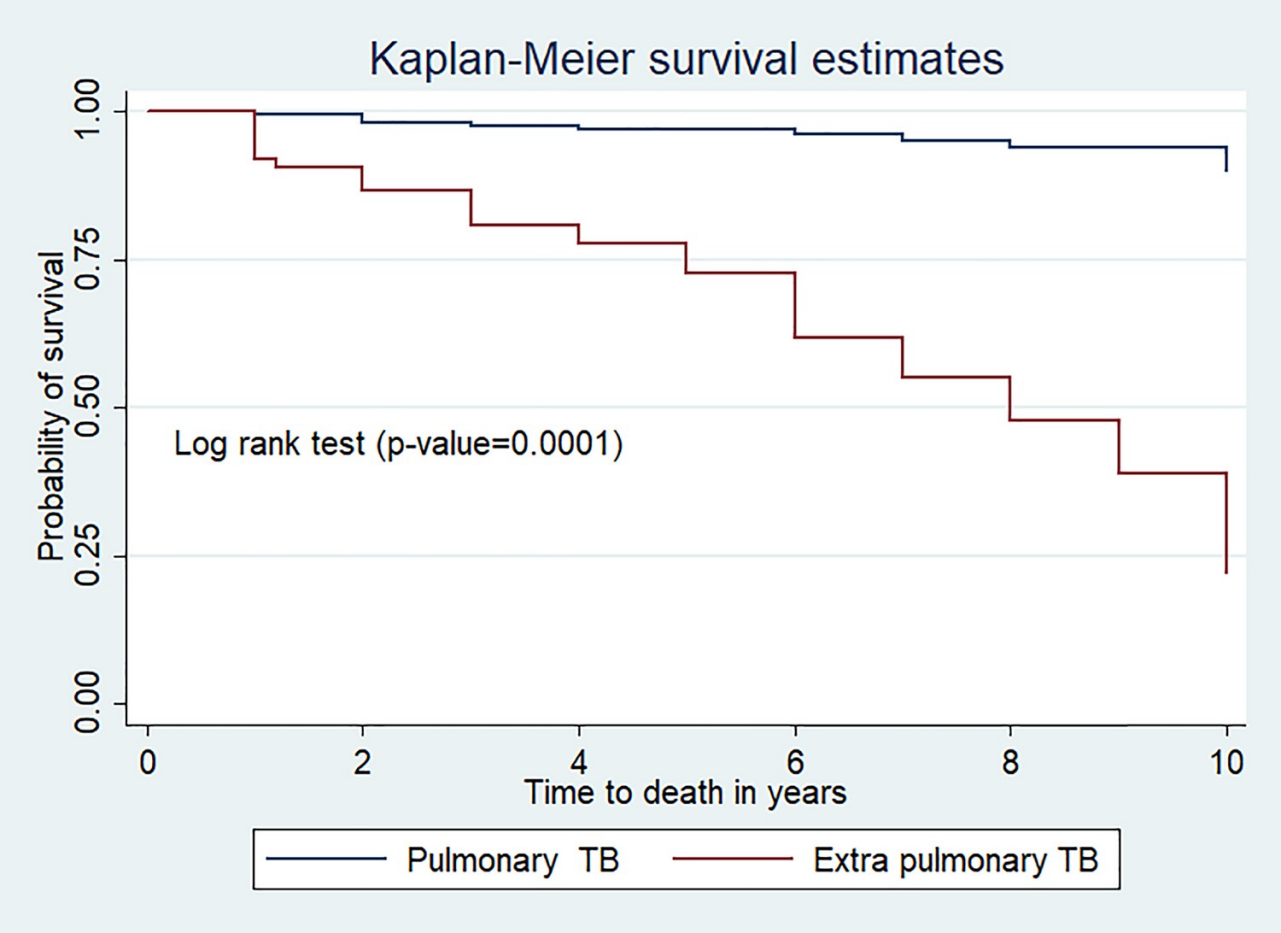
Kaplan Meier survival curve of infection among TB/HIV co-infected children at public hospitals, Southern Ethiopia.

The mean survival time of children with pulmonary TB was 9.68(95%CI; 9.44, 9.91) year and it was 7.12 (95%CI; 6.38, 7.87) years for those with extrapulmonary TB (p-value<0.0001) (**Fig 5**).

### Predictors of mortality among TB/HIV co-infected children

The bivariate analysis result showed that hemoglobin level, initiation of cotrimoxazole preventive therapy (CPT), initiation of Isoniazid prophylaxis (IPT), type of tuberculosis (TB), and adherence to ART drugs were candidate variables for the time to death among TB/HIV co-infected children. On the other hand; initiation of IPT, type of TB, adherence to ART drugs and hemoglobin level were statistically significant in multivariable cox proportional hazard model.

Hemoglobin level was statistically significant in the multivariable Cox proportional hazard model. The risk of mortality among TB/HIV co-infected children who had hemoglobin levels less than 10g/dl was 2.6 times higher as compared with those children who had hemoglobin levels≥10g/dl (AHR: 2.6; 95%CI: 1.28, 5.21). Children who had not initiated Isoniazid prophylaxis (IPT) had a 2.8 times higher risk of mortality as compared with the counterparts who initiate IPT (AHR:2.8; 95%CI:1.44,5.48).

The risk of mortality among TB/HIV co-infected children who had developed extra pulmonary or/and disseminated tuberculosis was 5.7 times higher as compared with those who had developed pulmonary tuberculosis (AHR: 5.7; 95%CI: 2.67, 12.56). TB/HIV co-infected children who were not adhered to ART drugs had5.2 times higher risk of mortality as compared with the counterparts, who had adhered to the ART drugs (AHR: 5.2; 95%CI: 2.19, 12.39) (**Table 6**).

**Table 6:** Predictors of mortality among TB/HIV Co-infected children at public hospitals in Southern Ethiopia, 2020.

Variables	Category	Survival status		95% CI		P-value
		Dead n = 47	Censored n = 227	CHR(95%CI)	AHR(95%CI)	
Age (year)	<1	2	9	0.84(0.19,3.59)		
	1–5	4	14	0.98(0.33,2.85)		0.77
	6–10	18	95	0.72(0.38,1.34)		
	11–14	23	109	1		
Sex	Male	22	117	0.99(0.56,1.77)		0.99
	Female	25	110	1		
Health service utilization	Primary hospital	6	43	0.62(0.26,1.48)		0.29
	General Hospital	41	184	1		
Residence	Urban	32	180	1		
	Rural	15	15	1.77(0.96,3.29)		0.067
Baseline WHO	Mild	18	91	1		
	Advanced	29	136	1.34(0.74,2.43)		0.31
Hgb at TB diagnose	<10 mg/dl	27	17	9.69(5.36,17.53)	**2.6(1.28,5.21)**	0.008
	> = 10 mg/dl	20	210	1	1	
CPT	Yes	20	199	1	1	0.7
	No	27	28	6.27(3.5,11.89)	1.14(0.57,2.27)	
IPT	Yes	16	189	1	1	0.002
	No	31	38	7.11(3.88,13.04)	**2.8(1.44,5.48)**	
Type of TB	PTB	9	178	1	1	
	EPTB	38	49	11.86(5.72,24.57)	**5.7(2.67,12.56)**	0.0001
Adherence	Adherence	9	184	1	1	
	Non-adherence	38	43	13.43(6.48,27.83)	**5.2(2.19,12.39)**	0.0001

## Discussion

The multivariable Cox proportional hazard model revealed that anemia, isoniazid preventive therapy initiation, extra-pulmonary tuberculosis, and non-adherence to ART were independent predictors of mortality among TB/HIV co-infected children.

The incidence density of TB/HIV co-infected children in this study was 2.97(95%CI; 2.2, 3.9) per 100 Child-year of follow-up which was slightly higher than a study conducted in Nigeria which was 1.4per 100 Child-year follow-up) [1]. It was slightly consistent with the study conducted at Northwest Ethiopia, which was 3.27 per 100 child-year of follow-up [2]. Such incidence difference might be due to the difference in the follow-up period and study setting. The incidence rate in this study was 17.15% which was almost similar to the study conducted in South Africa and India which was 17.5% and 17% respectively [1, 16]. This might be explained by the similarity in WHO HIV/AIDS implementation strategy, treatment, care, and support strategy. This finding was slightly higher than the study conducted in Northwest Ethiopia, which was 14.02% [2]. This might be due to the variation in the type of care provision across institutions.

However, the incidence of mortality in this study was lower than a study conducted in Thailand and India which was 30% and 36.5 respectively [16, 17]. This discrepancy might be due to the difference in study period and setting. The cumulative survival rate at the end of the follow-up period was 56%. This finding was lower than the study conducted in Nigeria, which was 73%. This discrepancy in survival rate might be due to the difference in the follow-up period of the studies.

Consistent with the study conducted at Tanzania and Malawi [18, 19], the risk of mortality among TB/HIV co-infected children who had diagnosed anemia was two times higher as compared with the counterparts, who had no diagnosed anemia (AHR: 2.6; 95%CI: 1.28, 5.21). This is due to the effect of anemia on the oxygen intake capacity which had a synergistic effect with tuberculosis and HIV co-infections that increase the prognosis of the disease process which may end up with death [2]. In addition; it might be associated with the decrease in hemoglobin level (commonly lower than 10mg/dl) due to the occurrence of pediatric infections [20].

The initiation of isoniazid preventive therapy had a protective effect against death among TB/HIV co-infected children. In line with the study conducted at South Africa and Nigeria [21, 22], this study revealed that, children who did not initiate isoniazid preventive therapy had two times higher risk of mortality as compared with the counterparts who initiated isoniazid preventive therapy (AHR: 2.8; 95%CI: 1.44, 5.48). This is due to the fact, IPT decreases mycobacterium load and reduces progression of latent bacilli to active TB [2].

Consistent with the study conducted at Gondar Comprehensive Specialized hospital and the United States of America and Thailand [2, 17, 23], the risk of mortality among TB/HIV co-infected children who had extrapulmonary tuberculosis was five times higher as compared with those who had pulmonary tuberculosis (AHR: 5.7; 95%CI: 2.67, 12.56). Extra pulmonary TB especially the disseminated one was more severe than pulmonary TB because which resulted in delayed recognition and which had hematogenous dissemination, finally which increases the mortality rate [2, 24].

In line with the study conducted at Northwest Ethiopia, India, and Addis Ababa [2, 24, 25], the risk of mortality among TB/HIV co-infected children who had non-adherence to ART drugs were five times higher as compared with those who had adherence to ART drug (AHR: 5.2; 95%CI: 2.19, 12.39). This indicates the beneficiary effect of adherence to ART because ART improves the immune status of the children and reduces the risk of mortality. In addition; when the child adheres to ART, viral replication was suppressed. This suppression of viral replication results from an increase in CD4 cells and increases the survival of children with TB/HIV co-infection. On the other hand, children who were not adhered to ART became at risk for treatment failure, which finally leads to death [2, 26].

The limitations of this study was, since it was conducted through record review certain variables which were not recorded such as viral load, family monthly income and drug abuse status of the parents were not analyzed.

## Conclusion

The mortality rate was high among TB/HIV co-infected children at public hospitals in southern Ethiopia. Extra-pulmonary tuberculosis, anemia, non-adherence to ART and initiation of isoniazid preventive therapy was an independent predictor of mortality among TB/HIV co-infected children. Therefore, children with extra pulmonary tuberculosis and anemia should be closely monitored to increase their adherence as well as they should initiate isoniazid preventive therapy.

## Supporting information

S1 Data (DTA)

## Abbreviations

ART: Antiretroviral therapy
AOR: Adjusted odd ratio
ART: antiretroviral therapy
CI: Confidence interval
HIV: Human immunodeficiency virus
IDR: Incidence density rate
OIs: Opportunistic infections: Tuberculosis
TB/HIV: Human immunodeficiency virus and Tuberculosis co-infection
